# Excessive Prenatal Supplementation of Iodine and Fetal Goiter: Report of Two Cases Using Three-dimensional Ultrasound and Magnetic Resonance Imaging

**DOI:** 10.1055/s-0041-1729143

**Published:** 2021-05-12

**Authors:** Pedro Castro, Heron Werner, Paulo Roberto Silva Marinho, Ana Paula Matos, Pedro Pires, Edward Araujo Júnior

**Affiliations:** 1 Department of Fetal Medicine, Clínica de Diagnóstico por Imagem, Rio de Janeiro, RJ, Brazil; 2 Department of Radiology, Universidade Federal do Rio de Janeiro, Rio de Janeiro, RJ, Brazil; 3 Department of Obstetrics and Gynecology, Universidade Federal do Estado do Rio de Janeiro, Rio de Janeiro, RJ, Brazil; 4 Department of Obstetrics and Gynecology, Universidade de Pernambuco, Recife, PE, Brazil; 5 Department of Obstetrics, Escola Paulista de Medicina, Universidade Federal de São Paulo, São Paulo, SP, Brazil; 6 Medical course, Universidade Municipal de São Caetano do Sul, São Paulo, SP, Brazil

**Keywords:** fetal goiter, prenatal care, iodine supplementation, three-dimensional ultrasound, magnetic resonance imaging, bócio fetal, atenção pré-natal, suplementação de iodo, ultrassonografia tridimensional, ressonância magnética

## Abstract

Fetal thyroid complications in pregnancy are uncommon, and are commonly related to the passage of substances through the placenta. The excessive iodine intake during the pregnancy is a well-known mechanism of fetal thyroid enlargement or goiter, and invasive procedures have been proposed for the treatment of fetal thyroid pathologies. In the present report, we demonstrate two cases from different centers of prenatal diagnosis of fetal thyroid enlargement and/or goiter in three fetuses (one pair of twins, wherein both fetuses were affected, and one singleton pregnancy). The anamnesis revealed the ingestion of iodine by the patients, prescribed from inadequate vitamin supplementation. In both cases, the cessation of iodine supplement intake resulted in a marked reduction of the volume of the fetal thyroid glands, demonstrating that conservative treatment may be an option in those cases. Also, clinicians must be aware that patients may be exposed to harmful dosages or substances during pregnancy.

## Resumo

As complicações fetais da tireoide na gravidez são incomuns e são comumente relacionadas à passagem de substâncias pela placenta. A ingestão excessiva de iodo durante a gravidez é um mecanismo bem conhecido de aumento da tireoide ou bócio fetal, e procedimentos invasivos foram propostos para o tratamento de patologias da tireoide fetal. No presente relato de caso, demonstramos dois casos de diferentes centros de diagnóstico pré-natal de aumento da tireoide fetal e/ou bócio em três fetos (um par de gêmeos, em que ambos os fetos foram afetados, e uma gravidez única). A anamnese revelou a ingestão de iodo pelos pacientes prescrita por suplementação inadequada de vitaminas. Nos dois casos, a interrupção da ingestão de suplemento de iodo resultou em uma redução acentuada do volume das glândulas tireoides fetais, demonstrando que o tratamento conservador pode ser uma opção nestes casos. Além disso, os médicos devem estar cientes de que as pacientes podem ser expostas a doses ou substâncias nocivas durante a gravidez.

## Introduction

The thyroid gland is essential for the neurodevelopment in the embryo and in the fetus; fetal thyroid pathologies can cause perinatal complications ranging from preterm delivery and airway obstruction to severe psychomotor impairment in childhood. 1 The incidence of fetal thyroid complications during pregnancy is of ∼ 1 per 4,000 live births, and it is usually related to the passage through the placenta of substances ingested by the mother during pregnancy or by antibodies acting on the fetal thyroid tissue. 2 Excessive maternal iodine intake during pregnancy is a well-known mechanism of fetal thyroid disease. High iodine exposure can lead to the development of fetal hypothyroidism and fetal goiter. 3 Among fetuses with congenital hypothyroidism, only 3% manifest fetal goiter, with a prevalence of 1 per 40.000 live births. 1 4 Fetal thyroid enlargement or goiter can be diagnosed based on nomograms of the thyroid circumference related to the gestational age, or of its biparietal diameter. 1 In the present case report, we describe two cases of three fetuses (one pair of twins, wherein both fetuses were affected, and one singleton pregnancy) with fetal thyroid enlargement and goiter caused by excessive maternal iodine exposure during pregnancy from unprescribed supplements.

## Case Report

**Fig. 1:**
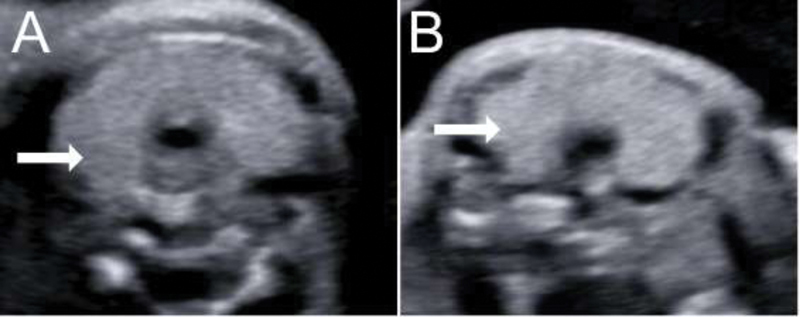
Two-dimensional ultrasonography of fetal thyroid gland (arrows) at the 28 ^th^ ( **A** ) and at the 31 ^st^ week ( **B** ). The reduction in volume is visible in a few weeks.

The first case is a primiparous 27-year-old pregnant woman who presented with polyhydramnios at the 22 ^nd^ week of the pregnancy. At the 28 ^th^ week, a hyperechoic thyroid gland trespassing the 98 ^th^ centile was observed ( Fig. 1 ). Esophageal pouch sign was observed, and the polyhydramnios persisted. During anamnesis, the patient reported the use of vitamin supplements, including iodine, with unknown daily dosage. The patient was informed of the possibility of iatrogenic fetal goiter, and she spontaneously interrupted the intake of vitamins. After 1 week, the patient presented normal thyroid function tests performed 1 week before, and the size of the fetal thyroid gland reduced to 28 mm × 7 mm; however, the polyhydramnios and the pouch sign persisted. At the 31 ^st^ week, the thyroid gland measured 25 mm × 5 mm, the amniotic fluid was normal, the pouch sign was invisible, and the amniotic fluid was normal even at the 35 ^th^ week.

**Fig. 2:**
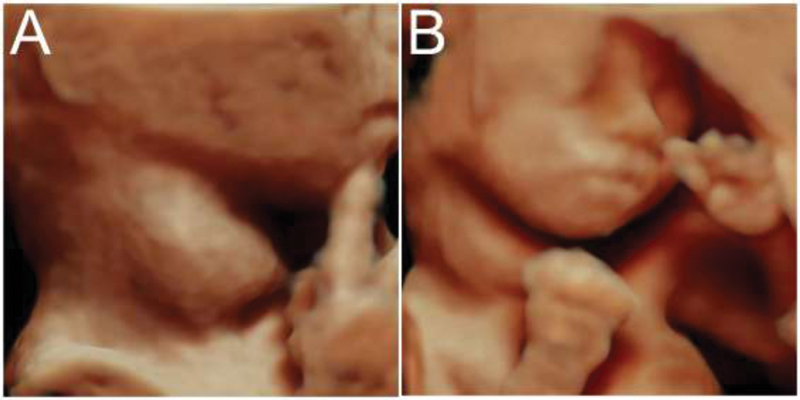
Three-dimensional ultrasonography in the rendering mode reconstruction of fetus 2 at the 22 ^nd^ week showing enlarged thyroid gland.

**Fig. 3:**
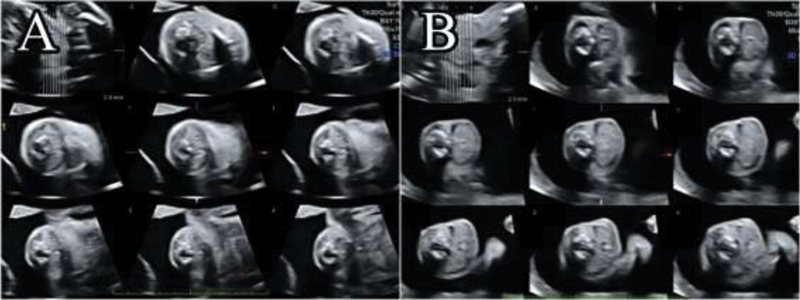
Three-dimensional ultrasound in the tomographic ultrasound imaging mode of the thyroid gland of fetus 1 ( **A** ) and fetus 2 ( **B** ) at the 22 ^nd^ week, showing disproportion and enlargement of the thyroid gland of fetus 2.

**Fig. 4:**
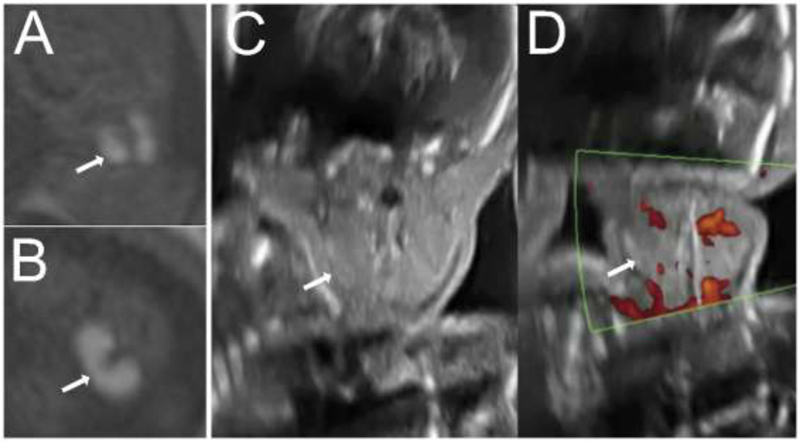
Fetus 2 at 22 weeks. Magnetic resonance imaging T1-weighted sequences of the neck showing the hyperintense signal of the thyroid gland (A, coronal; and B, axial), ultrasound images of the thyroid gland with and without color Doppler (C and D).

**Fig. 5:**
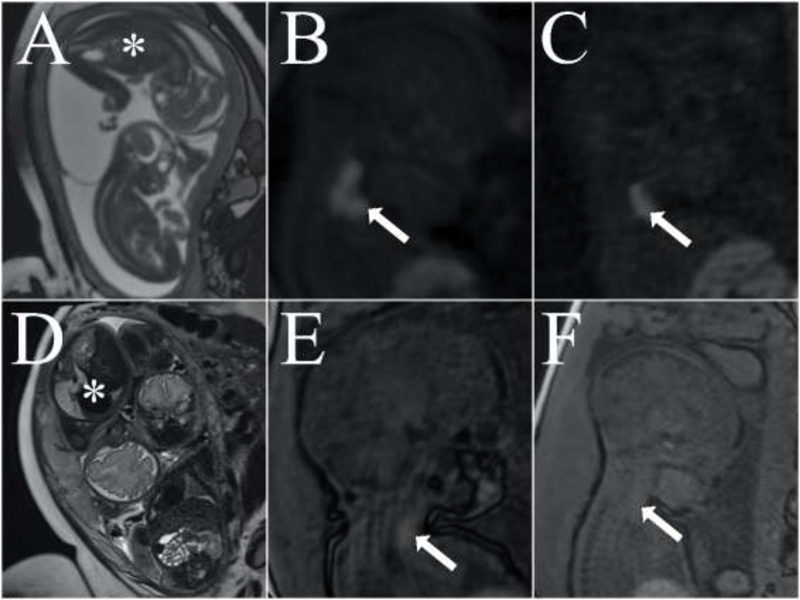
A, B, and C: Magnetic resonance imaging (MRI) at the 28 ^th^ week. A: T2-weighted sequence showing fetus 2 (asterisk) in polyhydramnios. B: T1-weighted sequence showing enlarged thyroid gland with hyperintense signal in fetus 2. C: The hyperintense signal is present in fetus 1, but with the thyroid gland presenting normal dimensions. D, E, and F: MRI performed at the 33 ^rd^ week. D: the amniotic fluid volume remained normal (fetus 2, asterisk). E and F: Fetuses with thyroid glands presenting normal dimensions and intensity compared with images B and C.

The second case is a 32-year-old woman G1P0, presenting with a dichorionic diamniotic pregnancy and an enlarged hyperechogenic mass on the anterior neck of fetus 2 in the 22-week scan ( Figs. 2 , 3 and 4 ). When inquired about the use of iodine supplementation, the patient mentioned the regular use of vitamin supplements with individualized dosages, prescribed by a lay person. In the same week, magnetic resonance imaging (MRI) was performed, which revealed hyperintense thyroid glands in both fetuses on T1-weighted sequence. Polyhydramnios was present, but there were no signs of esophageal compression. The patient was advised to discontinue the supplement intake, to which she promptly agreed. Also, the thyroid function was evaluated, with normal results. Fetal ultrasound examination performed at the 25 ^th^ week demonstrated a reduction in the dimensions of both thyroid glands, and the MRI demonstrated normal intensity in both thyroid glands on the T1-weighted sequence at the 33 ^rd^ week ( Fig. 5 ).

## Discussion

Fetal goiter is an unusual finding in prenatal ultrasound screening. As an essential element required for the synthesis of thyroid hormones, iodine is concentrated in the thyroid gland, and it crosses the placenta via active transport for fetal utilization. However, excessive iodine exposure can cause fetal goiter and thyroid dysfunction. 5

In the end of the last century, studies reported the effects of subclinical hypothyroidism and neurocognitive development of offspring, thereby increasing the attention toward supplementation and research on the thyroid gland during pregnancy. 6 7 The World Health Organization (WHO) has recommended the assessment of urinary iodine concentration (UIC) in pregnant and lactating women, a new standard to assess the iodine nutrition status. A UIC level < 150 μg/L is considered as an insufficient iodine status, between150 and 249 μg/L as adequate, between 250 and 499 μg/L as more than adequate, and > 500 μg/L as excessive. Furthermore, different guidelines recommend an increase in daily iodine intake to 250 μg during pregnancy and lactation. 8 However, iodine supplementation can have other effects on pregnancy outcomes. A study including 9,245 pregnant women in the 1 ^st^ trimester conducted in an iodine-sufficient region concluded that iodine supplementation in pregnancy must not exceed the UIC value of 250 μg/L. Values above this limit significantly increased the risk of developing subclinical hypothyroidism, and a significantly high risk of hypothyroxinemia was observed in pregnant women when the UIC was > 500 μg/L. 9

Excessive iodine intake inhibits thyroid hormone secretion and thyroid biosynthesis in healthy individuals in an acute period. After a prolonged excessive iodine intake, the thyroid function returns to normal levels. This phenomenon is known as escape from Wolff–Chaikoff effect and protects against the overproduction of thyroid hormones after iodine overexposure. However, some individuals, including fetuses, do not escape these effects and develop acquired isolated hypothyroxinemia. The immature fetal and neonatal thyroid glands are unable to reduce the intracellular iodine transportation; although excessive iodine is available, hypothyroidism persists. This hypothyroidism can resolve spontaneously after the cessation of excessive iodine intake. 10

The effects of fetal thyroid disease have been well described. Normal neurodevelopment requires an adequate amount of maternal thyroid hormones during the 1 ^st^ half and the 2 ^nd^ half of pregnancy by the maternal and fetal thyroids. Increase in this hormonal production demands the doubling of pregestational requirements of iodine intake. During the 1 ^st^ half of the pregnancy, human chorionic hormone acts like a thyroid stimulating hormone (TSH). Its molecular activity is proportionally very low compared with that of TSH; however, its concentrations during the 1 ^st^ trimester are sufficient to inhibit the pituitary secretion of TSH. At between 18 and 22 weeks of gestation, the pituitary-portal vascular system is fully developed, and the fetus is able to produce thyroid hormones. 11 The concentration of thyroid hormones and the activity of deiodinases have been investigated in different areas of the fetal brain. 12 These hormones have a particular action in the neurogenesis and in the migration of radial neurons in the developing cortex and cerebellum, changing and adding complexity in their cytoarchitecture even during the neonatal life. 11

With several etiologies, including congenital dyshormonogenesis, excess or deficiency of iodine, and others, fetal goiter is a less frequent consequence of fetal hypothyroidism, but increases fetal and neonatal morbidity due to congenital thyroid dysfunctions. The direct impact of thyroid masses can lead to obstruction of the airways and of the esophagus, increasing the risk of preterm birth from polyhydramnios and requiring a multidisciplinary team to secure the neonatal airway through the ex-utero intrapartum treatment procedure. The compressive effect on the airway and on the esophagus is a predictable complication, wherein MRI has an important role in the assessment of soft tissues and to characterize the permeability of the airway. Magnetic resonance imaging has also been used for virtual reconstruction of the airways and delivery planning in cervical masses. 13

In the cases presented here, identification of the possible cause of fetal goiter and cessation of supplemental ingestion led to conservative management with posterior regression of the goiter in both singleton and twin pregnancies. A previous study also described the regression of fetal hypothyroidism in twin pregnancy, or goiter, caused by excessive iodine intake. 14

Intra-amniotic levothyroxine injections have been proposed for the treatment of fetal goiter and hypothyroidism based on evidence suggesting that treatment delay increases the risk for impaired development in the infant. 15 Intra-amniotic therapy presents good results in progressive and dysfunctional goiters, decreasing the risk of perinatal complications. However, evidence indicating the benefits on neurodevelopment outcomes is scarce and controversial. The evident reduction in thyroid volume after the cessation of iodine supplements in our cases associated with the risk of invasive procedures guided the decision to follow-up the pregnancy conservatively. Unfortunately, the effects of the thyroid function on the long-term outcomes of the neonates are unknown, as well as the results of the thyroid function tests after delivery.

## Conclusion

In conclusion, we reported a case of a twin pregnancy affected by fetal goiter in both fetuses, which was induced by an excessive intake of iodine supplementation during the pregnancy, with successful regression after supplement suspension. Excessive iodine intake should be investigated as a possible cause of fetal goiter.
